# Phenolic compounds versus SARS-CoV-2: An update on the main findings against COVID-19

**DOI:** 10.1016/j.heliyon.2022.e10702

**Published:** 2022-09-20

**Authors:** Vicente Amirpasha Tirado-Kulieva, Ernesto Hernández-Martínez, Tania Jakeline Choque-Rivera

**Affiliations:** a Facultad de Ingeniería de Industrias Alimentarias y Biotecnología, Universidad Nacional de Frontera, Sullana, Piura, Peru; b Facultad de Ingeniería, Universidad Nacional de Jaén, Jaén, Cajamarca, Peru; c Facultad de Procesos Industriales, Universidad Nacional de Juliaca, Juliaca, Puno, Peru

**Keywords:** Polyphenols, SARS-CoV-2, COVID-19, Coronavirus, Medicine natural, Immune system

## Abstract

The COVID-19 pandemic caused by SARS-CoV-2 remains an international concern. Although there are drugs to fight it, new natural alternatives such as polyphenols are essential due to their antioxidant activity and high antiviral potential. In this context, this review reports the main findings on the effect of phenolic compounds (PCs) against SARS-CoV-2 virus. First, the proven activity of PCs against different human viruses is briefly detailed, which serves as a starting point to study their anti-COVID-19 potential. SARS-CoV-2 targets (its proteins) are defined. Findings from *in silico*, *in vitro* and *in vivo* studies of a wide variety of phenolic compounds are shown, emphasizing their mechanism of action, which is fundamental for drug design. Furthermore, clinical trials have demonstrated the effectiveness of PCs in the prevention and as a possible therapeutic management against COVID-19. The results were complemented with information on the influence of polyphenols in strengthening/modulating the immune system. It is recommended to investigate compounds such as vitamins, minerals, alkaloids, triterpenes and fatty acids, and their synergistic use with PCs, many of which have been successful against SARS-CoV-2. Based on findings on other viruses, synergistic evaluation of PCs with accepted drugs against COVID-19 is also suggested. Other recommendations and limitations are also shown, which is useful for professionals involved in the development of efficient, safe and low-cost therapeutic strategies based on plant matrices rich in PCs. To the authors' knowledge, this manuscript is the first to evaluate the relationship between the antiviral and immunomodulatory (including anti-inflammatory and antioxidant effects) activity of PCs and their underlying mechanisms in relation to the fight against COVID-19. It is also of interest for the general population to be informed about the importance of consuming foods rich in bioactive compounds for their health benefits.

## Highlights

• Phenolic compounds are known for their high potential against various human viruses.
• Phenolic compounds also have anti-inflammatory and immunomodulatory activity.
• Medicinal plants used against COVID-19 are rich in phenolic compounds.
• Phenolic compounds interfere with the activity of SARS-CoV-2 proteins.
• A wide variety of food products with high polyphenolic content are presented.

## 1 Introduction

We are currently experiencing a global health crisis due to a new coronavirus originating in Wuhan, China in 2019. First, World Health Organization (WHO) named it 2019-nCoV, but subsequently and until today, it is known as Severe Acute Respiratory Syndrome Coronavirus 2 (SARS-CoV-2) (Piccolella et al., 2020). This virus generates the disease COVID-19, which infects animals and humans (Mani et al., 2020). COVID-19 is highly contagious and has high morbidity and mortality rates (El-Missiry et al., 2021). As of 27 April 2021, according to WHO (2021), there are 508,827,830 cases and 6,227,291 confirmed deaths. In addition, COVID-19 also affects in social and economic terms (Annunziata et al., 2020; Mani et al., 2020).

COVID-19 infection starts out appearing to be a cold and then worsens over time. Symptoms include sore throat and headache, fatigue, fever, dry cough, conjunctivitis, lung and gastrointestinal problems (Khalil and Tazeddinova, 2020). Damage to the kidney and nervous system was also reported (Russo et al., 2020). Symptoms are aggravated if the infected person is elderly, smokes and/or consumes alcohol in excess (Mehany et al., 2021), if they suffers from hypertension, diabetes, obesity, pulmonary and cardiovascular diseases (Das et al., 2021a) and mainly if the patient has a weak immune system. 20% of infected persons present severe symptoms (Santos et al., 2021).

The global threat posed by COVID-19 makes it necessary to develop multiple alternatives for its possible therapeutic management, as existing drugs have side effects on the body. Therefore, scientists continue to strive immeasurably in the search for more efficient methods.

**Figure 1:**
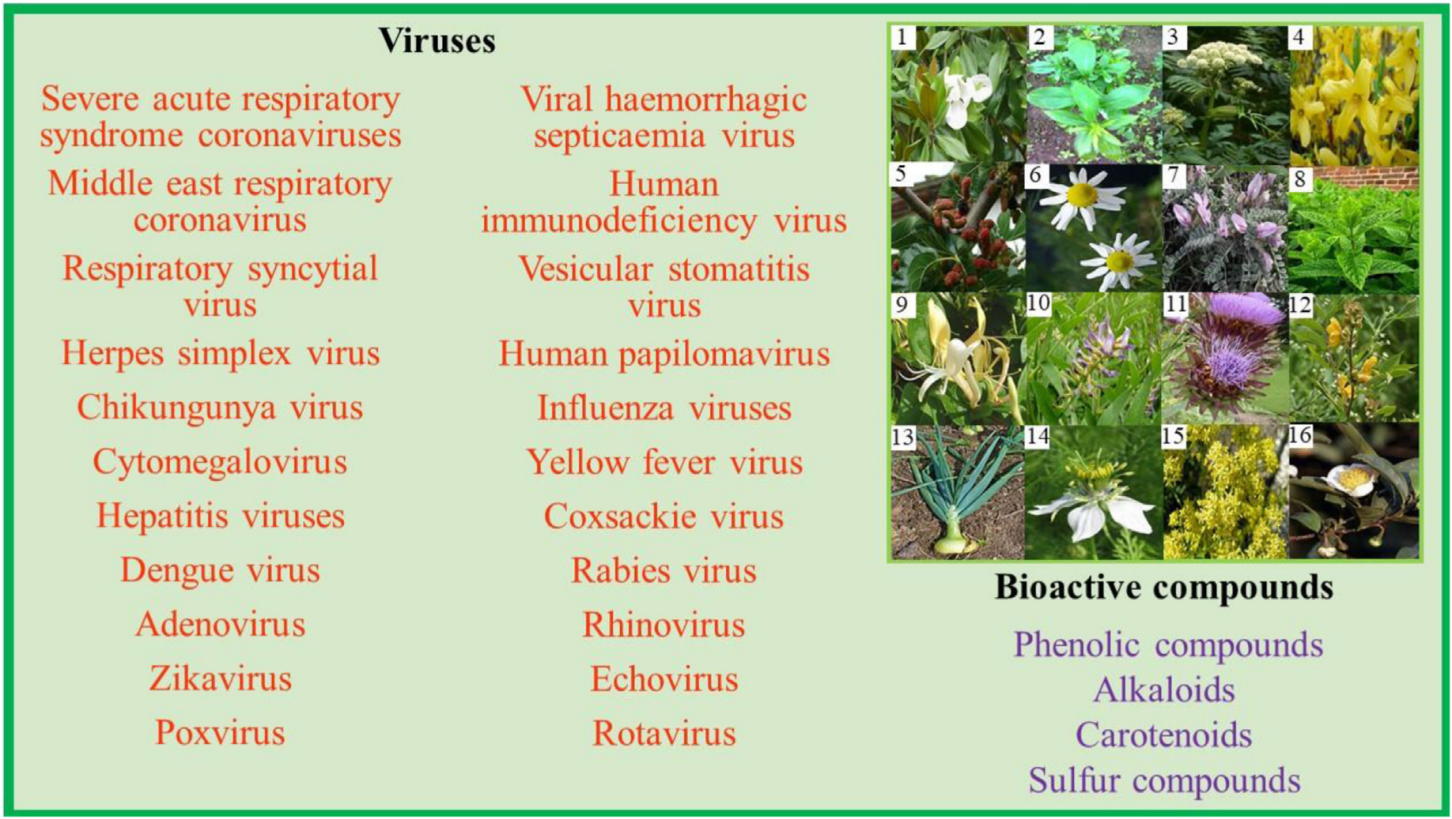
Bioactive compounds activity of some medicinal plants against various human viruses. 1) *Magnoliae officinalis*, 2) *Atractylodes macrocephala*, 3) *Angelicae dahuricae*, 4) *Forsythia suspensa*, 5) *Folium mori*, 6) *Matricaria recutita*, 7) *Astragalus membranaceus*, 8) *Herba menthae*, 9) *Lonicerae Japonicae Flos*, 10) *Glycyrrhiza glabra*, 11) *Cynara scolymus*, 12) *Cassia occidentalis*, 13) *Allium cepa L.*, 14) *Nigella sativa*, 15) *Isatis indigotica*, 16) *Camellia sinensis*. Design adapted from Adnan et al. (2021).

Medicinal plants have been used as natural drugs for many years due to their high bioactive content (Jalal et al., 2021). A wide variety of medicinal plants (leaf, steam, root, bark and fruit) have shown a high potential against various human viruses (Behl et al., 2021; Singh et al., 2021); however, their mechanism of action is still uncertain (Celik et al., 2021). In particular, natural medicine had optimal results in the treatment of people infected with SARS-CoV-1. In a study on the *in vitro* evaluation of extracts from more than 50 Chinese medicinal plants, Wen et al. (2011) determined that they have the necessary properties to be used as a therapy against SARS-CoV-1. *Atractylodes macrocephala*, *Magnoliae officinalis*, *Angelicae dahuricae*, *Forsythia suspensa* (Ho et al., 2020), *Folium mori*, *Flos chrysanthemi*, *Astragalus membranaceus*, *Herba menthae*, *Lonicerae Japonicae Flos* (Shahrajabian et al., 2020), *Glycyrrhiza glabra*, *Cynara scolymus*, *Cassia occidentalis*, among other medicinal plants, also showed excellent results when evaluated *in vitro* against SARS-CoV-1 (Patel et al., 2021). More details on the viruses studied are shown in Figure 1.

According to the results obtained and taking into account that SARS-CoV-1 and 2 have a high similarity between their sequence identities (79.5%) (Keflie and Biesalski, 2021) and their target proteins, it is postulated that natural medicine has a high potential to address current SARS-CoV-2. Since the spread of the disease in China, based on the experiences on the use of traditional herbal medicine in patients with Middle East Respiratory Syndrome Coronavirus (MERS) and SARS-CoV, this alternative therapy also started to be used in the possible therapeutic management of patients with SARS-CoV-2 (Alam et al., 2021; Yang et al., 2021). It has been promoted to prescribe mixtures of various Chinese medicinal plants (such as *qingfei paidu* decoction), which have been successful in the treatment of COVID-19 (Ren et al., 2020). In general, according to Russo et al. (2020), 92% effectiveness was obtained in all stages of COVID-19 and of the 5% of patients who continued with severe symptoms, none presented sequelae upon overcoming the disease. It was determined that the herbs used had a high phytochemical potential, highlighting the phenolic compounds (PCs) (Han et al., 2021). These results are confirmed by previous studies on other viruses treated in most Asian countries (Li and Peng, 2013). The extract of *Torreya nucifera* leaf showed a high anti-SARS-CoV-2 potential and when evaluating its bioactive content, the presence of four flavonoids (amentoflavone, quercetin, luteolin and apigenin) was highlighted (Chojnacka et al., 2020). It was determined that the presence of cinnamtannin B1, procyanidin A2 and procyanidin B1 in Cinnamomi Cortex extract conferred a high activity against SARS-CoV-1 (Russo et al., 2020). Similarly, in the study by Chiow et al. (2015), the antiviral potential of *Houttuynia cordata* Thunb. extract against dengue virus and mouse coronavirus (currently used as a model of COVID-19) was mainly attributed to the presence of quercetin, quercetrin and rutin.

PCs are natural compounds found in a wide variety of plant materials. PCs have unique characteristics and their consumption is reported to help prevent metabolic, cardiovascular, respiratory, neurological and cancerous diseases (Behl et al., 2021; Tirado-Kulieva et al., 2021). This is due to their high antimicrobial, antioxidant and anti-inflammatory activity (Tirado-Kulieva et al., 2022). PCs also modulate the immune system (de la Lastra et al., 2021; Mehany et al., 2021). Considering these characteristics and focusing on the antiviral property, Ali et al. (2022) indicate that most medicinal plants contain a high polyphenolic concentration. In the study by Katalinic et al. (2006), PCs content of the extracts of 70 medicinal plants ranged from 9 to 2218 mg catechin equivalent/L. This quality can be exploited to combat COVID-19. Table 1 shows some related studies.

**Table 1:** *In vitro* investigations on the polyphenolic potential of medicinal plants on different human viruses evaluated.

Reference	Plants	Compounds	Virus evaluated
Agrawal et al. (2020)	*Allium cepa L*.	Quercetin and quercetinglycosides	SARS-CoV-2
Luo et al. (2020)	*Fructus forsythia*, *Radix saposhnikoviae*, *R*. *glycyrrhizae*, *R. astragali*, *Rhizoma Atractylodis macrocephalae* and *Lonicerae Japonicae Flos*	Polyphenols	SARS-CoV-1
Ulasli et al. (2014)	*Anthemis hyalina* and *Nigella sativa*	Polyphenols	SARS-CoV-1
Siddiqui et al. (2020)	*Citrus aurantium*	Hydroxycinnamic acids and flavanone glycosides	SARS-CoV-1
	*Quercus infectoria* G.	Catechin, caffeine, resveratrol, p-coumaric, p-hydroxybenzoic acid, e-vanillic acid and 3-hydroxytyrosol cinnamic	SARS-CoV-1
Nolkemper et al. (2006)	*Salvia officinalis*, *Melissa officinalis*, *Rosmarinus officinalis*, *Mentha piperita*, *Thymus vulgaris* and *Prunella vulgaris*	Rosmarinic acid, apigenin rutinoside, luteolin, caffeic acid, hesperidin and eriodictyol	HSV-1 and HSV-2
Song et al. (2005)	*Camellia sinensis*	Epigallocatechin, epicatechin gallate and epigallocatechin gallate	A (H1N1) and A (H3N2)
Choi et al. (2009)	*Houttuynia cordata*	Quercetin 3-rhamnoside	Influenza A
Haidari et al. (2009)	*Punica granatum*	Punicalagin, ellagic acid, luteolin and caffeic acid	A (H1N1), A (H3N2) and influenza B
Sornpet et al. (2017)	*Psidium guajava*, *Andrographis paniculata*, *Gynostemma pentaphyllum*, *Curcuma longa* and *Kaempferia parviflora*	Polyphenols	Avian A (H5N1)
Miki et al. (2007)	*Ginkgo biloba*	Ginkgetin	A (H1N1) and A (H3N2)
Felipe et al. (2006)	*Guazuma ulmifolia* Lam. and *Stryphnodendron adstringens*	Catechin, gallocatechin, epicatechin and epigallocatechin	Poliovirus
Zuo et al. (2005)	*Saxifraga melanocentra* Franch	Polyphenols	HCV
Yamai et al. (2003)	*Glycine max* L. Merrill	Polyphenols	ADV and CV

Based on the findings reported in the literature, PCs are a suitable alternative to combat COVID-19. However, despite the importance of PCs, the field continues to grow steadily in this pandemic era. Moreover, it is difficult to fully define the relevance of PCs against SARS-CoV-2 due to the constant emergence of its new variants (such as omicron and delta). This is also a problem for vaccines, whose limits are remarkable (Snoussi et al., 2021). Therefore, it is necessary to update the data with new perspectives to support the development of appropriate therapeutic strategies by professionals related to the field. In this sense, this review will expand the knowledge on the anti-COVID-19 potential of PCs with recent evidence. First, some findings that corroborate the antiviral potential of PCs will be briefly shown, whose evidence is the basis for their study in the current context. Subsequently, the use of medicinal plants and, specifically, of more than 50 PCs isolated from different sources to counteract SARS-CoV-2 will be highlighted, defining the mechanism of action against its structural and non-structural proteins. The immunomodulatory role of PCs will also be defined, highlighting the mechanism of action. Finally, some limitations will be defined and an analysis of future trends will be carried out.

## 2 Methods

The bibliographic search was performed in the Scopus database in April 2022. After several attempts and discussion among the authors of this study, the methodology of Jalali et al. (2021) was adapted, presenting the main findings in three sections:

A. First, the findings on the antiviral activity of PCs and plant matrices rich in PCs were determined. The following search string was used: (“phenolic compounds” OR phenols OR polyphenols) AND (antiviral OR viruses). An amount of 5742 documents were found. All types of articles (mainly reviews) published since 2020 and presenting results of *in silico, in vitro* and *in vivo* studies were included. The information presented was brief and the purpose was to determine the high antiviral potential of PCs. 13 articles were selected for this section.
B. Specifically, this section focused on the potential of PCs and plant matrices rich in PCs as prevention and possible therapeutic management against COVID-19 and the underlying mechanisms. The first search string was modified: (“phenolic compounds” OR phenols OR polyphenols) AND (coronavirus OR COVID-19 OR SARS-CoV-2). An amount of 587 documents were found. *In vitro* and *in silico* studies and clinical trials were included. No *in vivo* studies on the effect of PCs on SARS-CoV-2 targets were found. 47 articles were selected for this section. During the writing of the manuscript, the snowballing technique was used to include other studies relevant to the topic (from any period) from the references of the selected articles. Since the targets of SARS-CoV 1 and 2 are very similar, results on activity against SARS-CoV were also shown, as suggested by Gligorijevic et al. (2021).
C. To complement the anti-COVID-19 potential of PCs, a brief explanation was made on their immunomodulatory/anti-inflammatory/antioxidant effect, which strengthens the body's defenses to prevent COVID-19 or resist its impact in case of infection. The same search string was used as in section B, but only 7 articles on *in vitro* and *in vivo* studies were included. The snowballing technique was also carried out. Some findings on this biological activity of PCs were also found in the selected articles on clinical trials in Section B.

### 2.1 Antiviral potential of polyphenols

PCs are plant secondary metabolites whose function is to defend plants against stressful situations, pathogens and environmental factors (Annunziata et al., 2020; Russo et al., 2020). They are composed of aromatic rings (phenyl) linked to hydroxyl groups (OH) and are classified into flavonoids (the most numerous group), tannins, xanthones, phenolic acids, lignans, among others. Further information on the classification and metabolism of PCs, the polyphenolic composition of foods and the effect of processing can be found at http://phenol-explorer.eu, a specialized database on the topic.

PCs have demonstrated broad antiviral spectrum against herpes simplex virus type 1 (HSV-1), hepatitis A (HAV), hepatitis B (HBV) and hepatitis C virus (HCV), influenza A (H1N1) virus and human immunodeficiency virus (HIV) (Paraiso et al., 2020). Studies on the use PCs to combat enterovirus (EV) (Annunziata et al., 2020), respiratory syncytial virus (RSV) (Iddir et al., 2020), chandipura virus (CHPV), japanese encephalitis virus (JEV) and SARS-CoV-2 was reported in the literature (Das et al., 2021a). According to Mehany et al. (2021), although the effect of PCs is concentration-dependent, the proportions used are low.

According to *in silico, in vitro* and *in vivo* research, polyphenols interfere in the different stages of viral infection. PCs act from the entry of the virus into the host cell, until its intracellular replication, release and dysfunction of the organism by the virus (Umeoguaju et al., 2021). In this sense, quercetin was able to inhibit HSV-1 and RSV activity, epigallocatechin-3-gallate was efficient against HBV, HSV-1 and adenovirus (ADV), and apigenin, pelargonidin and procyanidin against HSV-1 (Iddir et al., 2020). Similarly, theaflavins showed effects against A (H1N1), HSV-1, picornavirus type 1 (PV-1), coxsackievirus (CV) and rotavirus (RTV) (Mehany et al., 2021). Catechins inhibited HIV and HSV activity, baicalein was efficient against EV and dengue virus (DV), kaempferol was effective against HSV-1 and HSV-2 and myricetin against HBV and SARS-CoV-1 (Behl et al., 2021). Curcumin has been used to combat influenza virus, hepatitis viruses, HSV, HIV, chikungunya virus (HKV) and SARS-CoV-1 (Singh et al., 2021). Tannins were efficient against EV, HSV and SARS-CoV-2 (Das et al., 2021a), quercetin against DV, A (H1N1) and rhinovirus (RNV), and resveratrol against RNV, RSV and MERS-CoV (Khalil and Tazeddinova, 2020). Epicatechin, naringenin-7-O-glucoside and isorhamnetin-3-O-rutoside had good results against HSV (Khan et al., 2021), apigenin against picornavirus (PV) (Russo et al., 2020), caffeic acid against HCV (Dahab et al., 2020), and epigallocatechin gallate (EGCG) against human coronaviruses HCoV-OC43 and HCoV-229E (Jang et al., 2021).

### 2.2 Anti-COVID-19 potential of polyphenols

#### 2.2.1 Key SARS-CoV-2 targets

PCs act on the “key components” of SARS-CoV-2, its structural proteins. The function of the spike (S) is related to host cell penetration, the membrane (M) helps to develop the virus capsid and assemble its structure, the envelope (E) packages the virus, and the nucleocapsid (N) contains the packaged viral genome (Das et al., 2021a). PCs also act on the non-structural proteins of SARS-CoV-2. Some key targets are 3-chymotrypsin-like protease (3CLpro) or main protease (Mpro) (Patel et al., 2021), papain-like protease (PLpro), helicase, nucleoside-triphosphatase (NTPase) and N7-methyltransferase (N7-MTase) (Piccolella et al., 2020; Russo et al., 2020).

#### 2.2.2 *In silico* and *in vitro* evidence

The high mutation and recombination of SARS-CoV-2 is a serious problem for drugs that act on the aforementioned proteins (Keflie and Biesalski, 2021). In this context, PCs also target viral receptors such as angiotensin-converting enzyme 2 (ACE2) (Paraiso et al., 2020), which is immediately recognized by the virus due to the S protein. This usually generates the S protein-ACE2 complex, 20-fold more likely compared to the S protein of SARS-CoV-1 (Das, 2020). Curcumin modulates ACE2 levels, preventing the alteration in the organism of infected people (Paraiso et al., 2020). Thymol from thyme essential oil and oregano essential oil was also reported to be efficient in inhibiting the activity of S protein (Kulkarni et al., 2020). Emodin from *Rheum officinale* roots interfered at the S protein-ACE2 interface, the reason for which was competition at the recognition binding domain (RBD) of the S protein (Zhou et al., 2020). Curcumin from *Curcuma longa*, epigallocatechin gallate from tea, herbacetin from *Rhodiola spp*., PCs from *Citrus spp*., piceatannol and resveratrol from berries also showed a strong interaction with S protein, interfering with its binding to ACE2 (Yamamoto et al., 2016; Das et al., 2021a, 2021b; Tallei et al., 2020; Singh et al., 2021).

Specific areas of proteins are also investigated such as the protease domain (PD) of ACE2, the RBD (which binds to PD) of S protein (Albohy et al., 2020), S1 (contains the RBD) and S2 (responsible for membrane fusion subsequent to the role of S1) subunit protein (Harisna et al., 2021; Verma et al., 2020), and the protease enzyme 6LU7, which is the crystal structure of the 3CLpro (Sherif et al., 2021). Arokiyaraj et al. (2020) determined that kaempferol, kaempferol 7-o-rhamnoside, quercetin, kaempferitrin, geraniin, corilagin, protocatechuic acid, gallic acid and ellagic acid are compounds with the potential to inhibit the RBD activity of S protein of the SARS-CoV-2. A large number of flavonoid glycosides, ellagitannins and stilbenoids showed a strong interaction with the human transmembrane serine protease (TMPRSS2), preventing its role in viral and host cell membrane fusion by cleaving at the S1/S2 and S'2 sites (Puttaswamy et al., 2020).

Naringenin, kaempferol and quercetin from citrus fruits (Tutunchi et al., 2020), eckol, 7-phloroeckol, fucodiphloroechol-G and phlorofucofuroeckol A from algal (Al-khafaji et al., 2021), peonidin 3-O-sophoroside, isopeonidin 3-O-sambubioside and protocatechuic acid 4-O-glucoside from black tea and berries (Bhatia et al., 2020), and pelargonidin from *Pimpinella anisum* (Mehany et al., 2021) inhibited 3CLpro activity of SARS-CoV 1 and 2. Similar results were shown when evaluating luteolin, genistein, coumaric acid, syringic acid, gallic acid, chlorogenic acid, vanillic acid, daidzein, chrysin, hesperetin, galangin, eriodictyol, polydatin and neobavaisofavone from rosemary, thyme, basil, oregano, mint and sage (Khalil and Tazeddinova, 2020), and 2,2-dimethyl-8-prenylchromene, artepillin C, p-coumaric acid, caffeic acid phenethyl ester, ellagic acid, hesperetin, naringenin, kaempferol, quercetin and chrysin from honey and propolis (Shaldam et al., 2021), and 12 vanillin derivatives (Law et al., 2020). Inhibition of 3CLpro is crucial because it plays a key role in virus transcription and replication (Mehany et al., 2021). Due to its importance, it is the main target for the study and development of drugs with the potential to inhibit coronaviruses (Badraoui et al., 2022).

PLpro influences virus replication and infection. In addition, its inhibition would prevent the production of other proteins such as nsP1, nsP2 and nsP3, agents that also act in viral replication (Verma et al., 2020). Fortunately, many isolated PCs such as baicalein, hesperidin, quercetin, luteolin, gallocatechin, epigallocatechin gallate, kaempferol, and isoliquiritigenin have shown efficiency in its inhibition (Paraiso et al., 2020; de la Lastra et al., 2021). For example, xanthoangelol E from *Angelica keiskei*, PCs from *Psoralea corylifolia* seeds and PCs from *Broussonetia papyrifera* showed an IC50 value of de 1,2, 15 g/L y 3,7 μM, respectively against SARS-CoV PLpro (Gligorijevic et al., 2021). When evaluating the potential of myricetin from *Scutellaria baicalensis* against nsP13 helicase of SARS-CoV, the IC50 value was 2.71 μM. With this result, the authors determined that the entry of the virus into the cell was blocked (Llivisaca-contreras et al., 2021). The same value showed myricetin from *Chondropetalum mucronatum* (Verma et al., 2020).

Others targets of interest are the non-structural protein such as nsP13 (Zia et al., 2021), nsP16 (Albohy et al., 2020) and RNA-dependent RNA polymerase (RdRp) (Verma et al., 2020). Inhibition of RdRp is necessary because its role is to catalyze virus RNA replication (Verma et al., 2020). Resveratrol, epigallocatechin, quercetagetin, and myricetin showed strong affinity for RdRp, successfully interfering with virus replication (Paraiso et al., 2020) and slowing the progression of COVID-19 in patients, regardless of the severity of the infection. Selvaraj et al. (2021) analyzed the affinity of 30 phytochemicals from *Plectranthus amboinicus* against the RdRp of SARS-CoV-2. The best results were obtained with some PCs such as rutin, luteolin, rosmarinic acid and salvianolic acid. Similarly, the effect of 12 PCs of honey and propolis against the RdRp of SARS-CoV-2 was evaluated. Quercetin, kaempferol, ellagic acid and p-coumaric acid had the highest affinity/inhibition (Shaldam et al., 2021). Quercetin, myricetin, apigenin, chrysin, chlorogenic acid and ellagic acid from *Moringa oleifera* showed high inhibition potential against nsP9 and nsP10 (Muhammad et al., 2021). PCs such as curcumin, rosmarinic acid and ursolic acid showed strong affinity for nsP15 of SARS-CoV-2 (Kumar et al., 2020).

In *in vitro* studies, the IC50 value is useful for determining the concentration of PCs required to inhibit 50% of the activity of SARS-CoV-1 and 2 targets (Table 2). In addition, most studies are performed *in silico*, i.e. by simulation or computational analysis. Molecular docking is the most widely used technique as it helps to predict the relationship between a protein and compounds based on affinity or binding energy (Vardhan and Sahoo, 2021). In this case, the impact/affinity of PCs on the active region of SARS-CoV-2 proteins is evaluated. Lower binding energy means higher efficiency (Xu and Chang, 2007). The advantages of conducting research using simulation processes based on existing data are that it is less time consuming and significantly reduces costs. For example, Das et al. (2021b) rapidly evaluated the effect of 4 commercial drugs, 17 natural compounds, 2 antifungal drugs, 4 antiviral drugs, and 6 antinematodal and antiprotozoal drugs on the 3CLpro activity of SARS-Cov-2. It was concluded that rutin (−9.55 kcal/mol) had the highest docking score. Table 3 presents the main *in silico* findings, classifying PCs as promising compounds to combat the current pandemic.

**Table 2:** *In vitro* studies on the effect of PCs against different SARS-CoV-1 (^a^) and SARS-CoV-2 (^b^) targets.

Reference	Source	Compound	Target proteins	IC50 value
Annunziata et al. (2020)	N.S.	Gallocatechin gallate and catechin gallate	N protein^a^	500 μg/L (40% inhibition)
Lee et al. (2009)	N.S.	Quercetin	NTPase/helicase^a^	8.1 μM
Llivisaca-contreras et al. (2021)	*Scutellaria baicalensis*	Myricetin	nsP13 helicase^a^	2.71 μM
Mani et al. (2020)	*Broussonetia papyrifera*	3′-(3-methylbut-2-enyl)-3′,4,7-trihydroxyflavane	PLpro^a^	3.7 μM
Piccolella et al. (2020)	*Psoralea corylifolia L*. seeds	Psoralidin	PLpro^a^	4.2 μM
	*Psoralea corylifolia L*. seeds	Isobavachalcone	PLpro^a^	7.3 μM
	*Psoralea corylifolia L*. seeds	4′-O-methylbavachalcone	PLpro^a^	10.1 μM
	*Psoralea corylifolia L*. seeds	Bavachinin	PLpro^a^	38.4 μM
	*Betula ​papyrifera* roots	Papyriflavonol A	PLpro^a^	3.7 μM
	*Angelica keskei* leaves	Xanthoangelol E	PLpro^a^	1.2 μM
	*Angelica keskei* leaves	Coumarins	PLpro^a^	>200 μM
Siddiqui et al. (2020)	*Paulownia tomentosa* fruit	Tomentin E	PLpro^a^	5 μM
Mehany et al. (2021)	*Torreya nucifera* leaves	Luteolin	3CLpro^a^	20.2 μM
	*Torreya nucifera* leaves	Apigenin	3CLpro^a^	280.8 μM
	Brown algae	Dieckol	3CLpro^a^	2.7 μM
	*Isatis indigotica* roots	Sinigrin	3CLpro^b^	217 μM
	*Isatis indigotica* roots	Hesperetin	3CLpro^b^	8.3 μM
	*Torreya nucifera* leaves	Quercetin	3CLpro∗	23.8 μM
Park et al. (2013)	*Ecklonia cava*	Phlorotannins	3CLpro^a^	2.7 to >200 μM
Russo et al. (2020)	N.S.	Quercetin-3-β-galactoside	3CLpro^a^	42.79 μM
	*Torreya nucifera* leaves	Amentoflavone	3CLpro^a^	8.3 μM
	N.S.	Tomentin A-E	3CLpro^a^	5.0–14.4 μM
Paraiso et al. (2020)	*Rheum officinale* roots	Emodin	S protein-ACE2^a^	200 μM
Yu et al. (2012)	*Aglaia perviridis*	Myricetin	nsP13 ATPase^a^	2.71 μM
	*Aglaia perviridis*	Scutellarein	nsP13 ATPase^a^	0.86 μM

N.S.: Not specified.

**Table 3:** *In silico* investigations by molecular docking on the effect of PCs against different SARS-CoV-2 targets.

Reference	Source	Compound	Target proteins	Binding energy (kcal/mol)	Interaction of PCs with amino acid residues of virus targets.
Al-khafaji et al. (2021)	Algal	Dieckol	RBD/ACE2	−7.41	ACE2: (Gly354 and Ala386)^a^. RBD: (Lys390, Asp393, Gln396 and Tyr491)^a^.
		8,8-Bieckol	RBD/ACE2	−7.25	ACE2: Arg393^a^, (Thr324 and Gly354)^b^, and (Ala386 and Ala387)^c^. RBD: (Arg395, Gly490, Asp392, Gly391 and Gly490)^a^, Ile489^c^, (Arg395 and Asp392)^d^.
		6,6-Bieckol	S protein/TMPRSS2	−9.18	S protein: (Thr827, Val826 and Asn824)^a^. TMPRSS2: (Phe194, Pro288 and Pro354)^c^.
		Phlorofucofuroeckol B	S protein/TMPRSS2	−8.52	TMPRSS2: (Phe357, Cys241, Thr287 and Glu289)^a^, Pro288^b^, (Ala243, Pro288, Pro354 and Phe357)^c^, and Arg240^d^.
Albohy et al. (2020)	*Ocimum menthifolium*	Apigenin-7-O-rutinoside	3CLpro	−9.1	N.S.
		Didymin	3CLpro	−8.6	N.S.
		Prunin	nsP16/10	−9.3	N.S.
		Acaciin	nsP16/10	−9.4	N.S.
		Isosakuranetin	ACE2-PD	−7.7	N.S.
		Acacetin	ACE2-PD	−7.6	N.S.
		Salvitin	ACE2-PD	−7.5	N.S.
		Isosakuranin	RBD-S protein	−6.9	N.S.
Attia et al. (2021)	Orange peel	Hesperidin	3CLpro	−8.84	Thr 24^a^, (Thr 25, His41, Leu27, Asn119, Cys145, Ser123, Leu141 and Gly143)^e^, (Met 49 and Tyr118)^f^.
Bhatia et al. (2020)	Black tea	Theaflavin 3-O-gallate	3CLpro	−9,80	His163^a^, (Leu141, His41, Thr25, Met49, Met165, Glu166, His164, and Gln189)^e^, and Cys145^g^.
	Pomegranate	Punicalagin	3CLpro	−9.80	(Lys5, Phe140 and Glu288)^a^, and (Lys137, Glu288, Leu141, Leu286, Glu166, Glu290, Gly170, Phe140, and Ser139)^e^.
	Blackberry	Protocatechuic acid 4-O-glucoside	3CLpro	−9.80	Ser144^a^, and (Leu141, Gln189, Met165, Gly143, Glu166, His41, His163, Asn142, Cys145, and Asp187)^e^.
Abd El-Mordy et al. (2020)	*Mimusops laurifolia*	Rutin	3CLpro	−8.21	(Asn142 and Glu166)^a^.
		Mearnsitrin III	3CLpro	−7.59	(Phe140, Gln189 and Met49)^a^.
		Quercetin 3-O-α-L-rhamnopyranoside	3CLpro	−6.99	(Met165, Met49, Cys145, Gln189 and His 163)^a^.
Gentile et al. (2020)	*Ecklonia cava*	8,8′-Bieckol	3CLpro	−13.7	(His41 and Cys145)^a^.
		Dieckol	3CLpro	−12.0	(Leu27, Met41, Met49, Met165 and Leu167)^c^.
Ghosh et al. (2020)	*Camellia sinensis*	Epigallocatechin	3CLpro	−7.0	(Ser144, His163 and Gln192)^a^, (Ala191, Leu141, Glu166 and Met165)^c^, and (Ser46 and Asn142)^h^.
		Gallocatechin	3CLpro	−7.1	(Phe140, Glu166, Arg188 and Gln192)^a^, (Gln189, Met49, Met165 and Leu141)^c^ and (Ser46 and Asn142)^h^.
		Catechin	3CLpro	−7.1	(Leu141, Gln192, Ser144 and His163)^a^, (Leu27 and Met49)^c^ and (Ser46, Thr24, Thr25 and Thr45)^h^.
		Catechin gallate	3CLpro	−7.2	(Ser144, His163, Arg188 and Thr190)^a^, (Phe140, Leu141, Met165, Glu166 and His172)^c^ and Gln189^h^.
Harisna et al. (2021)	Propolis	3′ -Methoxydaidzin	3CLpro	−7.7	(Arg188, Glu166, Thr190 and Thr26)^a^ and Gln189^e^.
		Genistin	3CLpro	−7.6	(Arg188, Gln192, Glu166, Thr190 and Thr26)^a^ and Gln189^e^.
		Neobavaisoflavone	3CLpro	−7.6	Tyr54^a^, and (Met165, Cys145 and Met49)^f^.
		Methylophiopogonone_A	S2 protein	−8.2	(Lys1191, Arg1185, Asn1194, Ser939 and Ser940)^a^.
Kulkarni et al. (2020)	Thyme essential oil	Thymol	S RBD	−5.4	N.S.
	Oregano essential oil	Carvacrol	S RBD	−5.2	Ser459^a^.
	Eucalyptus essential oil	Eucalyptol	S RBD	−4.3	N.S.
Arokiyaraj et al. (2020)	Geranii Herba	Geraniin	S RBD	−7.58	(Leu441, Thr345, Asn450, Asp442, Arg346, and Ser349)^a^.
		Kaempferitrin	S RBD	−5.98	(Tyr451, Thr345, Phe347, Leu441, Arg509, Asp442, and Asn450)^a^.
		Kaempferol 7-O-rhamnoside	S RBD	−5.69	(Asn450, Thr345, Phe347 and Ser349)^a^.
Mhatre et al. (2021)	Green tea	Epigallocatechin gallate	3CLpro (6LU7)	−8.3	(Tyr54 and Glu166)^a^, (Glu166, Met49 and Cys145)^c^ and (Gly142, Gln189, Arg188, Asn142, Pro52, His41, Leu27, Leu141, Met165, Val42, Thr25, Thr26, Asp187, His163, and His164)^h^.
			S RBD	−9.7	(Trp47, Tyr91, Gln96, Arg408 and Ser35)^a^, (Ser94, Val50, Arg403, Lys417, Trp47, and Gly416)^c^ and (Gly92, Thr415, Tyr32, Tyr33, Tyr52, Tyr58, Tyr453, Tyr495, Glu406, Gln409, Asp95, and Asp97)^h^.
			PLpro	−8.9	(Gln269, Leu162, Gly160 and Asn109)^a^, (His89, Glu161 and Gly160)^c^ and (Val159, Asp108, Asn109, Cys270, Gly160, Glu161, Gln269, Gln269, and Leu162)^h^.
			RdRp	−5.7	(Glu610, Val609 and Ser607)^a^, (Pro612 and Lys603)^c^ and (Asp608, Thr806, Tyr826, Leu805 and Tyr606)^h^.
			ACE2 with S RBD	−8.5	(Ile291 and Lys441)^a^, (Ile291, Phe438, Met366, Leu370 and Ala413)^c^ and (Phe428, Asn437, Leu410, Leu418, Glu435, Gln442, Ser409, Asp367, Asp292, Glu430, Pro415, and Thr434)^h^.
	Black tea	Theaflavin digallate	3CLpro	−8.4	(Thr26, Thr24, Thr25, Phe140 and Glu166)^a^, (Met165, Met49, Cys145 and Thr26)^c^ and (Asn119, Asp187, Cys44, Ser46, Asn142, His41, His164, His172, Leu27, Leu141, Leu167, Arg188, Gly143, Gln189, Val186, and Thr45)^h^.
			S RBD	−11.6	(Ser94, Thr415, Tyr91, Asp95, Asp97, Asp61, and Gln414)^a^, (Lys417, Val50 and Arg403)^c^ and (Arg408, Lys64, Tyr32, Tyr33, Tyr52, Tyr58, Tyr453, Val98, Trp47, Gln409, Gly416, Gly92, Glu406, Ser35, Ser93, Asp405, Ala60, and Leu455)^h^.
			PLpro	−11.3	(Val159, Gln269, Thr158, Asn109, Leu162, and Glu161)^a^, (His89, Glu161 and Val159)^c^ and (Cys270, Gln269, Leu162, Gly160, Glu161, Thr158, His89, Asp108, Asn109, and Ser85)^h^.
			RdRp	-6.0	(Ser607, Val609 and Lys603)^a^ and (Pro612, Asp608, Leu805, Tyr606, Glu610, Thr604, and Lys751)^h^.
			ACE2 with S RBD	-8.0	Thr434^a^, (Leu370, Lys441, Ile291, Asp367, and Ala413)^c^ and (Thr445, Thr414, Thr276, Tyr279, Phe438, Met336, Asn290, Asn437, Asp292, Ser409, Dlu4435, Gln442, Glu406, and Pro415)^h^.
Allam et al. (2021)	*Sesamum indicum L*. seeds	Hydroxymatairesinol	PLpro	-7.21	(Gly143, Met165 and Ser144)^a^.
		Sesamin	PLpro	-6.55	(Thr190 and Gln189)^a^ and (Gln189 and His41)^f^.
		Sesamolin	RDRP	-6.98	(Thr 190 and Gln 189)^a^ and Gln 189^f^.
		Ferulic acid	RDRP	-5.37	(Thr 190 and Gln 189)^a^.
Mohammad et al. (2021)	*Livistona australis*	Genkwanin-6-C-beta-glucopyranoside	nsP10	−7.2	K4346^a^, (C4330 and R4331)^e^ and (C4330 and A4424)^f^.
Muhammad et al. (2021)	*Moringa oleifera*	Ellagic acid	nsP9	−7.1	(Ser47, Lys87 and Asp48)^a^ and (Asn28, Ser14 and Asp27)^e^.
		Chrysin	nsP9	−6.8	Ser6^a^ and (Leu5, Leu104, Ala108 and Leu113)^f^.
		Apigenin	nsP10	−7.1	(Cys4294 and Asn4293)^a^, Asn4358^b^, (Phe4321, Thr4292, Lys4296, Ala4357 and Gln4289)^e^ and Pro4290^f^.
Omotuyi et al. (2020)	*Aframomum melegueta*	Apigenin	3CLpro	−7–7	(C145, E166 and H163)^a^.
		Quercetin	S-Adenosylmethionine	−8.2	(F149, Y47, D130, G71 and D114)^a^.
		Tectochrysin	RBD/ACE2	−8.7	RBD: (Y505, Y495, D405, Y453, R403 and Q943)^a^. ACE2: (K353, D30, D38, E35, E37 and N33)^a^.
Sherif et al. (2021)	*Rhus spp*.	Methyl 3,4,5-trihydroxybenzoate	Protease enzyme 6LU7	−81.82	(His164, Leu141¸ Cys145, Ser144 and Phe140)^a^.
Suručić et al. (2021)	Pomegranate peel	Punicalin	S protein	−7.41	Ser371^a^ and (Val367 and Leu368)^f^.
			TMPRSS2	−8.17	(Asn97 and Arg405)^h^.
		Punicalagin	S protein	−7.31	(Asn370, Asn343 and Ser371)^a^ and (Leu335, Leu368 and Phe342)^f^.
			TMPRSS2	−7.36	(Arg 405 and Asn 97)^h^.
		Gallic acid	Furin	−7.49	(Asp258, Asp301, Asp306, Glu257, His194, Thr365, Thr262, Gly255, Pro256, Ser368, and Ser311)^h^.
Utomo et al. (2020)	*Citrus sp*.	Hesperidin	S protein	−9.61	N.S.
			RBD-ACE2	−9.50	N.S.
		Hesperetin	S protein	−9.08	N.S.
		Naringenin	PD of S protein	−12.44	N.S.
	*Curcuma sp*.	Bisdesmethylcurcumin	S protein	−8.64	N.S.
		Curcumin	RBD-ACE2	−9.04	N.S.
		Desmethylcurcumin	RBD-ACE2	−8.04	N.S.
	*Alpinia galanga*	Galangin	PD of S protein	−12.96	N.S.

Bonds/interactions: ^a^ Hydrogen, ^b^ Carbon-hydrogen, ^c^ Hydrophobic, ^d^ Electrostatic, ^e^ Van der Waals, ^f^ Pi, ^g^ Covalent, and ^h^ Polar.

N.S.: Not specified.

#### 2.2.3 Specific mechanisms of action

Based on PCs from algal, a general mechanism of action was described. First, PCs prevent the attachment and subsequent entry of the virus into host cells. If the virus manages to enter the organism, PCs inhibit the activity of its proteins (Al-khafaji et al., 2021). This prevents the recognition, multiplication and release of the virus (Wink, 2020). In addition, PCs such as flavonoids were reported to induce death in infected host cells (Wang et al., 2022).

**Figure 2:**
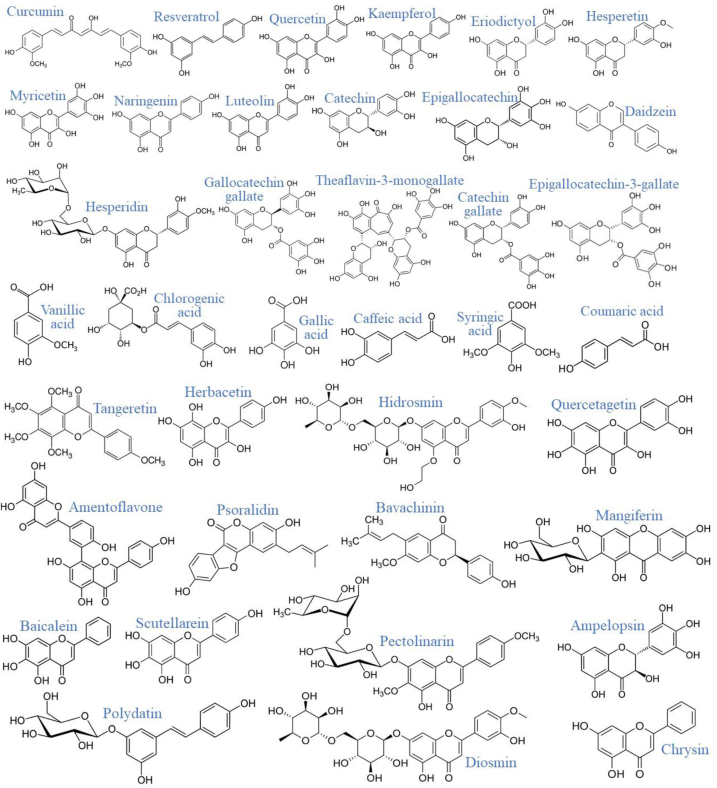
Chemical structure of most of the PCs mentioned in this study (Downloaded from ChemSpider, http://www.chemspider.com/).

PCs are efficient protein inactivators due to their interaction with phenyl rings (Tutunchi et al., 2020; de la Lastra et al., 2021), suitable for counteracting the effects of COVID-19 (Figure 2) (Chojnacka et al., 2020; El-Missiry et al., 2021).

Specifically, PCs interact with amino acid residues through hydrogen, electrostatic, and polar bonds, among others interactions (Table 3). The activity of the PCs depends on their structure. For example, phenolic acids from *Vitis amurensis* had a strong interaction with SARS-CoV-2 target residues due to their hydroxyl and carbonyl groups (Souid et al., 2022). Furthermore, the high biological activity of stilbenes is due to their two phenyl groups linked by a transethane bond; the biological activity of ellagic acid is due to its lipophilic domain (four phenolic groups) and mainly to its hydrophilic domain (four rings and two lactones); the anti-3CLpro potential of sotetsuflavone from *Dacrydium balansae* Brongn. & Gray is attributed to the position and number of the methyl groups (Puttaswamy et al., 2020).

A slight structural change is enough to affect the activity of the PCs. *In vitro*, Nguyen et al. (2021) determined that the greater the number of OH groups on the B-ring of flavonols, the greater the anti-COVID-19 activity: myricetin (three OH groups) > quercetin (two OH groups) > kaempferol (one OH group). It was also reported that glycosylation of quercetin and the OH group at position 7 of the A-ring of quercetagenin decreased their effect. In flavanones, the activity against 3CLpro of naringenin and hesperidin was attributed to glycosylation at position 7 of their A-ring. Hesperidin had less effect due to the methoxy group at position 5 of its B-ring. In flavan-3-ols and flavones, antiviral activity was also directly proportional to the OH groups in the B-ring. It was also determined that the presence of galloyl moiety at position 3 of the C-ring increased the effect of epigallocatechin, gallocatechin, epicatechin gallate and catechin gallate. In the case of flavones and isoflavones, biological activity was enhanced by glycosylation at position 8 of the A-ring. Finally, it was determined that the activity of diarylheptanoids on 3CLpro depends on the presence of methoxy groups: curcumin (two methoxy groups) > bisdemethoxycurcumin (no methoxy group) (Nguyen et al., 2021).

#### 2.2.4 Clinical trials

These drugs have had adverse effects, and their development and evaluation can take many years. National and international entities related to the field have joined forces with scientists and industry for the development of new and natural drugs (Tavakoli et al., 2022).

Supplementation with PCs has been successful as a method of COVID-19 prevention. 76 outpatients (18–80 years, 60.5% male) received two doses of 200 mg quercetin daily for 30 days. Among the findings, a) the number of patients hospitalized (9.2 vs 28.9%) was lower and for less time (1.6 vs 6.8 days) than in the control group (cg); b) the need for oxygen therapy was lower than in the cg (1,3 vs 19,7%); c) symptoms were not aggravated in any patient compared to the cg (10,5%) (Di Pierro et al., 2021a). Similarly, 120 outpatients (20–60 years, 52.5% male) received two doses of 250 mg quercetin daily for three months. The number of patients hospitalized (1,67 vs 6,67%) was lower than in the cg (Rondanelli et al., 2022). 53 outpatients (45–84 years, 45.3% male) received fourth doses of 500 mg resveratrol daily for 7–15 days. The number of hospitalized patients (2 vs 6%), visits to the emergency room (8 vs 14%) and the incidence of pneumonia (8 vs 16%) was lower than in the cg. There was also incidence of pulmonary embolism in the same proportion of patients in each group (2%) (Pawar et al., 2021).

To fully define the anti-COVID-19 potential of PCs, properly designed clinical trials are needed to ensure that the treatment is safe and effective (Nile and Kai, 2021). For example, Takdehghan et al. (2021) ruled out the toxicity of the extract of *Phoenix dactylifera L*. leaf (rich in PCs) by *in vitro* and *in vivo* assays in Wistar rats. Subsequently, they evaluated the effect of its intake in patients with COVID-19, obtaining optimal results. Likewise, frequent follow-ups should be carried out to detect possible side effects. Karimi et al. (2021) evaluated the effect of ingesting a polyherbal decoction rich in PCs. Promising results were obtained and at each visit any adverse effects were recorded and ruled out. Further findings on these clinical trials are shown in Table 4.

**Table 4:** Clinical trials on the evaluation of the administration of PCs or PCs-rich foods as a possible therapeutic management for patients with COVID-19.

Reference	Type of clinical trial	Sample (intervention group)	Severity of COVID-19	Treatment	Place and period	Main findings
PCs						
Ahmadi et al. (2021)	Controlled, randomized, triple-blind.	30 patients. Mean age: 41.3 years, 66.7% male.	Mild or moderate.	Nano-curcumin: 40 mg/6 times daily for 7 days.	Imam Reza Hospital, Iran. April to July 2020.	Improvement of symptoms except sore throat. Decreased levels of inflammatory markers. Increased lymphocyte count.
Asadirad et al. (2022)	Controlled, randomized, open-label.	30 patients. Mean age: 56 years, 20% male.	Mild or moderate.	Nano-curcumin: 240 mg daily for 7 days.	Razi Hospital, Iran. June to July 2020.	Reduced expression of proinflammatory cytokines.
Di Pierro et al. (2021b)	Controlled, randomized, open-label, still-oingoing.	21 patients. Mean age: 42.5 years, 47.6% male.	Mild.	Quercetin: 200 mg/3 times daily for 7 days and 2 times daily for 7 more days.	Department of Medicine, King Edward University, Pakistan. December 2020 to March 2021.	Higher number of recoveries than in the cg (16 vs. 2 in the first week, 5 vs. 17 in the second week). Decreased levels of inflammatory markers. No deaths, except in the cg (4.8% of patients).
Saber-Moghaddam et al. (2021)	Controlled, nonrandomized, open-label.	21 patients. Mean age: 53,5 years, 31,3% male.	Mild or moderate.	Nano-curcumin: 40 mg/2 times daily for 2 weeks.	Imam Reza and Quaem Hospitals, Iran. April to July 2020.	Improvement of symptoms (fever, chills, cough, myalgia, and tachypnea). Increased lymphocyte count. Need for supplemental O2 was 1,75 days shorter. Hospitalization was 4,1 days shorter. No deterioration of symptoms, except in the cg (40% of patients).
Shohan et al. (2022)	Controlled, randomized, open-label.	30 patients. Age: 35–75 years, 56.7% male.	–	Quercetin: 1 g daily for 7 days.	Ahvaz Razi Teaching Hospital, Iran. December 2020 to January 2021.	Decreased levels of inflammatory markers. Increased hemoglobin level. Increased respiratory rate. No deaths, except in the cg (10% of patients).
Valizadeh et al. (2020)	Controlled, randomized, double-blind	20 patients. Mean age: 53,3 years, 77% male.	–	Nano-curcumin: 40 mg/4 times daily for 14 days.	Imam Reza Hospital, Iran. Period not specified.	Decreased expression and secretion of inflammatory cytokines.
**Natural medicine rich in PCs (plants/substances/functional foods)**						
Al-haidari et al. (2021)	Controlled, randomized, open-label.	160 patients. Age: 13 to >65 years, 50% male.	–	*Nigella sativa*: 40 mg/kg daily for 13 days.	Kirkuk General Hospital, Irak. From September to November 2020.	Decrease in the severity of the infection. No deaths, except in the cg (5.4% of patients).
Ashraf et al. (2020)	Controlled, randomized, multicenter.	157 patients. Age: ≤ 40 to >80 years, 57.3% male.	Moderate or severe.	1 g/kg of honey plus 80 mg/kg of *N*. *sativa* daily for 13 days.	Four medical care facilities in Pakistan. April to July 2020.	Patients recovered with moderate (6 vs 10 days) and severe cases (8.6 vs 12 days) in less time than in the cg. No deaths, except in the cg (2,7% of patients).
Eskandarian et al. (2022)	Controlled, randomized.	34 patients Age: < 40 to >60 years, 67.6% male.	–	*Chlorella vulgaris* (300 mg/4 times daily) plus polyherbal brew^b^ (6 g/3 times daily) for 2 days minimum until the end of hospitalization.	Not specified.	Decreased levels of inflammatory markers. Decrease in the prevalence of diarrhea. Hospitalization was 2 days shorter.
Hashem-Dabaghian et al. (2021)	Controlled, randomized.	23 patients. Mean age: 36.6 years, 73.9% male.	Mild, moderate or severe.	Lavender syrup (5% of lavender extract plus 66,5% of honey plus 28,5% of water):9 mL/2 times daily for 3 weeks.	clinic of the Gonbad-E-Kavoos Health center, Iran. In 2020.	Improvement of olfactory dysfunction. Improvement of symptoms not significant.
Huseini et al. (2022)	Controlled, randomized, double-blind.	21 patients. Mean age: 58 years, 63.3% male.	–	Imfluna^c^ (102.48 mg GAE/g dry extract): about 500 mg/3 times daily for 14 days.	Baqiyatallah Hospital, Iran. May 2020.	Improvement of symptoms (cough and dyspnea). Decreased levels of inflammatory markers. Non-significant improvement of lung lesions.
Kamalinejad et al. (2021)	Controlled, randomized, double-blind.	25 patients. Mean age: 53.5 years, 36% male.	Mild or moderate.	Tiban^d^: 5 mL/3 times daily for 14 days.	Tertiary care center, Iran. April to May 2020.	Improvement of symptoms (dyspnea, fatigue, and appetite).
Karimi et al. (2021)	Controlled, randomized, open-label, multicenter.	182 patients. Mean age: 48.7 years, 58.1% male.	–	Polyherbal decoction^e^ (35.57 GAE/g): about 900 mL daily for 7 days.	Three hospitals in Tehran and two hospitals Isfahan, Iran. March to July 2020.	Improvement of symptoms (vertigo, muscle pain, dry cough, sputum cough, runny nose, chills, headache, anorexia, and fatigue). Duration of dyspnea was 2.3 days shorter.
Kosari et al. (2021)	Controlled, randomized.	25 patients. Mean age: 41 years, 48.7% male.	–	30 mL of syrup (1,5 mg of *Hyoscyamus niger* L. extract plus 450 mg of propolis)/daily for 6 days.	Akhavan and Sepehri Clinics, Iran. May to June 2020.	Improvement of symptoms (headache, dry cough, abdominal pain, chest pain, sore throat, fever, dyspnea, dizziness, and diarrhea).
Koshak et al. (2021)	Controlled, randomized, open-label, two-arm, parallel-group.	91 patients. Mean age: 35 years, 53% male.	Mild.	*N*. *sativa* oil: 500 mg/2 times daily for 10 days.	King Abdulaziz University Hospital, Saudi Arabia. May to September 2020.	Higher percentage of recovered patients (62 vs 36%) and in less time (10.7 vs 12.3 days) than in the cg.
Sardari et al. (2021)	Controlled, randomized.	21 patients. Mean age: 45.2 years, 42.2% male.	–	Thyme essential oil: 5 mL/3 times daily for 7 days.	Vali-e Asr Hospital, Iran. In 2020.	Improvement of symptoms (cough, fever, dyspnea, dizziness, cough, muscular pain, chest wall pain, headache, weakness, lethargy, fatigue, and anorexia). Increased lymphocyte count and reduced neutrophil count.
Shiri et al. (2021)	Controlled, randomized, double-blind.	21 patients. Mean age: 41 years, 51.4% male.	–	3 g of herbal supplement (1,5 mg of *Saccharum officinarum* plus 1 g of *Pistacia lentiscus*, and 0,5 g *Terminalia chebula* plus)/2 times daily The days of treatment are not specified.	Peymaniyeh Hospital, Iran. May to July 2020.	Improvement of symptoms (dyspnea, fever, cough, and myalgia). Patients recovered in less time (4.12 vs 8.37 days) than in the cg. No deaths, except in the cg (8.6% of patients).
Silveira et al. (2021)	Controlled, randomized, single center, open-label.	40 and 42 patients for the lowest and highest dose, respectively. Mean age: 49.5 years, 28% men for the lowest dose, and 48.9 years, 30% men for the highest male.	–	Propolis: 100 mg/4 times daily or 200 mg/4 times daily for 7 days.	São Rafael Hospital, Brazil. June to August 2020.	Patients recovered in less time (7 (lower dose) and 6 (higher dose) vs. 12 days) than in the cg. Decrease in acute kidney injury with higher dose.
Takdehghan et al. (2021)	Controlled, randomized, double-blind.	116 patients. Mean age: 52.2 years, 47.64% male.^a^	Mild or moderate.	*Phoenix dactylifera* L. leaf extract (28.2 mg GAE/g): 5 mL in 30 mL of water/5 times daily for 14 days.	Ganjavian Hospital, Iran. October to November 2020.	Decreased levels of inflammatory markers. Increased partial pressure of oxygen in the blood.
Tavakoli et al. (2022)	Controlled, randomized, single-blind, two-arm, parallel-group.	50 patients. Mean age: 56.8 years, 37% male.	Moderate.	Persian barley water (72 mg GAE/100 g dry matter): 250 mL daily for 14 days.	Ali Asghar Hospital, Iran. January to March 2021.	Decrease in fever. Decreased levels of inflammatory markers. Hospitalization was 4,5 days shorter.
Varnasseri et al. (2022)	Controlled, randomized, double-blind.	30 patients. Mean age: 47.9 years, 40% male.	–	*Phyllanthus emblica* (39.56 g GAE/100 g extract): 2 g of powder or 100 mL daily for 10 days.	Razi and Sina Hospitals, Iran. May to June 2020.	Patients recovered in less time (4.44 vs 7.18 days) than in the cg. Improvement of symptoms (fever, severity of cough, dyspnea, and myalgia.). Decreased levels of inflammatory markers. Decreased lung involvement.
Zhao et al. (2021)	Controlled, randomized, single center, open-label,	204 patients. Mean age: 52 years, 43.1% male.	Mild.	Huashibaidu granule^f^: 10 g/2 times daily for 7 days.	Dongxihu FangCang hospital, China. February to March 2020.	Symptoms worsened in fewer patients (2.5 vs 7.8%) than in the cg.

*Note:* All treatments were complementary to standard medication. Although it is emphasized that the natural medicine used is rich in PCs, it should be considered that they contain other bioactive phytochemicals such as carotenoids, terpenoids, lecithins, alkaloids, etc.

a age and percentage of male patients based on the total group (placebo and intervention).

b Mixture of *Althaea rosea*, *Mentha longifolia*, *Malva sylvestris*, and *Matricaria recutita*.

c Mixture of *Echinacea angustifolia* DC. aerial part, *Stachys lavandulifolia* Vahl aerial part, *Artemisia annua* L. aerial part, *Hyssopus officinalis* L. aerial part, *Polypodium vulgare* L. rhizome, *Alpinia officinarum* Hance rhizome, *Zingiber officinale* Roscoe rhizome, and *Panax ginseng* C.A.Mey root.

d Mixture of *Ziziphus jujuba* Mill and *Trachyspermum ammi* (L.) Sprague.

e Mixture of *Urtica dioica* L. leaf, *Althaea officinalis* L. flower, *Matricaria chamomilla* L. flower, *Nepeta bracteata* Benth. flower, *Zataria multiflora* Boiss. aerial part, *Glycyrrhiza glabra* L. root, *Ficus carica* L. fruit, and *Ziziphus jujuba* mill. fruit.

f Mixture of Herba Ephedrae, Armeniacae Semen Amarum, Gypsum Fibrosum, Glycyrrhizae Radix et Rhizoma, Pogostemonis Herba, Magnoliae Officinalis Cortex, Atractylodis Rhizoma, Tsaoko Fructus, Pinelliae Rhizoma Praeparatum, Poria, Rhei Radix et Rhizoma, Astragali Radix, Descurainiae Semen Lepidii Semen, and Paeoniae Radix Rubra.

### 2.3 Role of polyphenols in the immune system

#### 2.3.1 Effect of COVID-19 infection on the organism

For a good quality of life, it is essential to have an immune system in optimal condition. The immune system is related to physiological processes and defenses against microbial infections and other internal and external problems. A deficient immune system can lead to different diseases such as being more susceptible to contracting COVID-19 and presenting severe symptoms.

Innate immunity is activated when the organism is attacked by foreign agents. Since SARS-CoV-2 is extremely dangerous, the defense is so excessive that it causes overactivation of the NLRP3 inflammasome. This generates a severe storm of cytokines such as interferon-γ (IFN-γ), C–C chemokine ligand Motif 2 (CCL-2), CCL-3, tumor necrosis factor (TNF) and various interleukins (IL) (Cena and Chieppa, 2020). These cytokines act not only in the infected parts of the body, but also in healthy areas, causing irreparable damage to various organs (Ghati et al., 2021).

### 2.4 Fundamentals of the immunomodulatory activity of PCs

To stop the cytokine storm and avoid its lethality, the body must be kept in good condition and in a natural way. The necessary intake of certain dietary compounds helps modulate the immune system (Banik et al., 2021). PCs also have high immunomodulatory, anti-inflammatory and antioxidant activity, ideal for preventing damage to the immune system, maintaining homeostasis in the body and regulating energy metabolism to promote the absorption of nutrition (Wang et al., 2022).

PCs influence immune cells such as dendritic cells, lymphocytes, macrophages and leukocytes (Neyestani, 2008). Harisna et al. (2021) indicate that due to the phenolic potential of propolis, its consumption helps to increase the performance of macrophages. Specifically, kaempferitin, curcumin and quercetin are considered a potent immunostimulators as it acts on macrophages, splenocytes, natural killer (NK) cells and peripheral blood mononuclear cells (PBMC), which play a key role in immune function (Llivisaca-contreras et al., 2021; Ali et al., 2022). These cells play a role in the expression of cytokine genes. PCs influence the increase of anti-inflammatory cytokines (AICs) and the reduction of proinflammatory cytokines (PICs). For example, according to *in vitro* studies, curcumin has the potential to inhibit and/or control the production of PICs (Celik et al., 2021). Curcumin administration improved the condition of virus-infected mice, which was associated with suppression of cytokine storm (Roy et al., 2020).

### 2.5 *In vitroein vivo* studies

PCs such as catechins, resveratrol, genistein and vanillic acid induce the activation/deactivation of signaling pathways related to inflammation such as nuclear factor kappa B (NF-κB), Nuclear factor-erythroid factor 2-related factor (Nrf2), and Signal transducer and activator of transcription 1/3 (STAT1/3). This reduces PICs, chemokines, inducible nitric oxide synthase (iNOS) and cyclooxygenase (COX) (Khalil and Tazeddinova, 2020; Neyestani, 2008). Xanthohumol consumption reduced plasma IL-6 levels in mice by 80% through Nrf2 signaling (Miranda et al., 2016). Resveratrol increased the messenger RNA expression of AICs such as IL-10, and reduced that of PICs such as TNF-α, IL-2 and IFN-γ in mice by inhibiting MEK/ERK signaling pathway (Huang et al., 2020). Kaempferol helps decrease IL-1β and TNF-α expression by preventing NF-κB translocation (Khalil and Tazeddinova, 2020). Theaflavin from black tea and resveratrol also modulates the immune system by inducing the proper functioning of Mitogen activated protein kinase (MAPK) (Annunziata et al., 2020; Iddir et al., 2020). Chlorogenic acid significantly reduces NF-κB expression. This caused the reduction of PICs such as IL-12 and the increase of AICs such as IL-10 and IL-22, the latter also considered as PIC (Abaidullah et al., 2021). In mice, catechin ingestion induced inhibition of PI3K/AKT/mTOR signaling and increased T lymphocytes. This meant improvement in adaptive immunity and, in general, in attenuation of induced acute lung injury and oxidative stress (Yang et al., 2021). Naringin ingestion inhibited lipopolysaccharide-induced IL-1β, IL-6, iNOS and COX-2 production in a mouse model by inhibiting the expression of high mobility group box 1 (Cheng et al., 2020). Naringenin prevented the production of PICs in a murine model by inhibiting NF-κB translocation and MAPK phosphorylation (de la Lastra et al., 2021). Quercetin, kaempferol, myricetin, luteolin, baicalein and apigenin modulate the immune system by deactivating the NLRP3 inflammasome (Mckee et al., 2020). Similarly, quercetin reduced NLRP3 inflammasome activation in animal models (Ricordi et al., 2021).

The production of reactive oxygen species (ROS) is common in viral infections, and SARS-CoV-2 infection is no exception. PCs also prevent oxidative damage and amplification of the inflammatory response by ROS (Khan et al., 2019), helping to keep the immune system strong (Abedi et al., 2021). This is achieved by inhibiting ROS-producing enzymes and also by increasing the activity of antioxidant enzymes (El-Missiry et al., 2021). In this pandemic context, PCs such as lutein were reported to have high anti-inflammatory activity. de la Lastra et al. (2021) emphasize that luteolin can treat lung inflammatory disorders because it inhibits PICs and inflammatory enzymes. In addition, it prevents ROS production by suppressing signaling pathways such as NF-κB.

**Figure 3:**
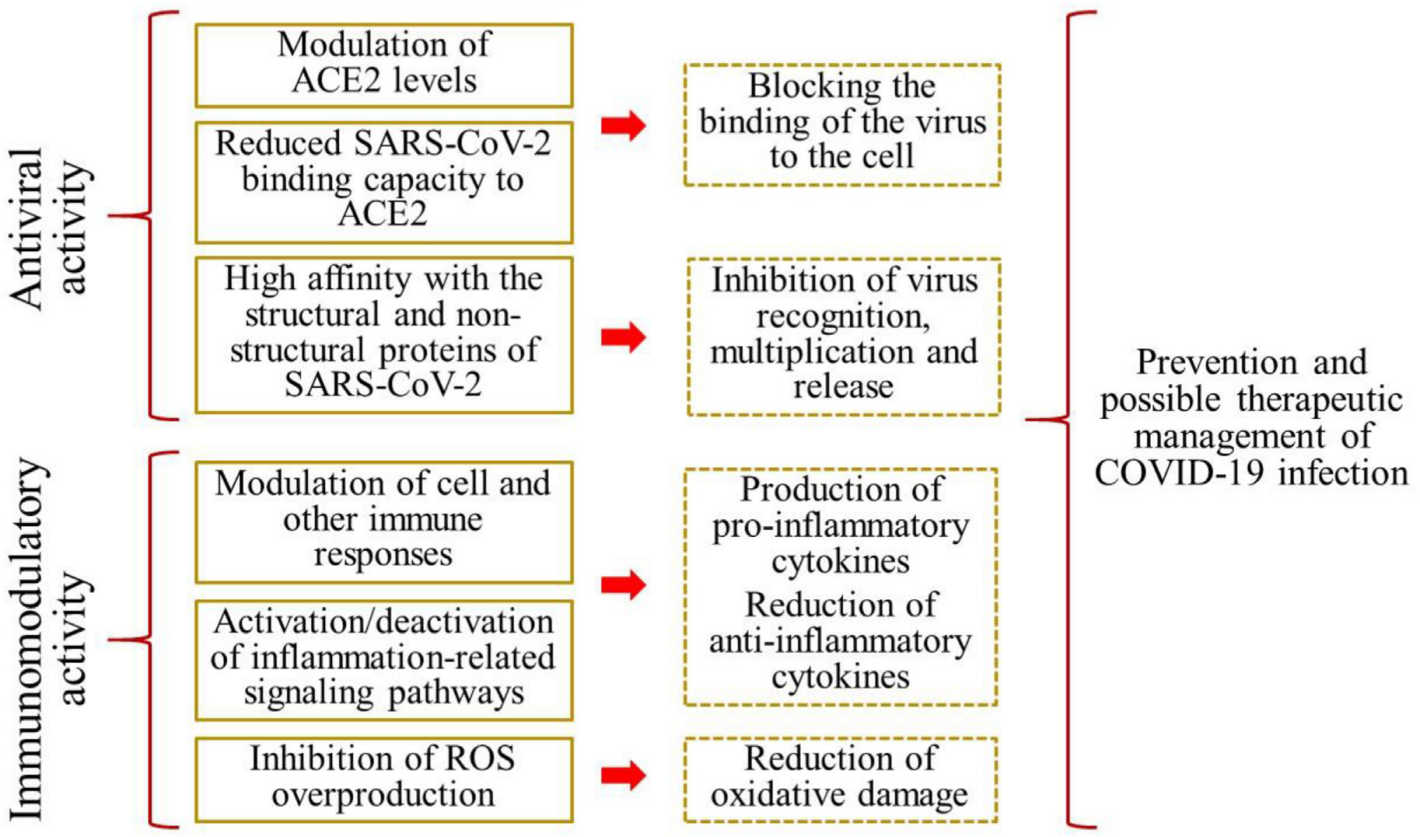
Representation of some proposed mechanisms of the anti-COVID-19 activity of polyphenols. Immunomodulatory activity encompasses the antioxidant and anti-inflammatory activity of PCs.

In summary, when the immune system is in good condition, viral infection would be stopped in the first phase (incubation stage). In this case, infected patients would present only mild symptoms (Khalil and Tazeddinova, 2020). Otherwise, if the person has a weak immune system, the infection will progress to the second stage and patients may experience severe symptoms. Likewise, although the mechanism of action of each specific polyphenol has not yet been defined, Figure 3 shows the anti-COVID-19 mechanisms of action of polyphenols in general.

### 2.6 Recommendations for future work

Studies on the effect of other compounds are recommended to broaden the range of options in the fight against COVID-19. In this sense, the evaluation of alkaloids (Ghosh et al., 2021), triterpenes (Abd El-Mordy et al., 2020), lignans, coumarins (Bhattacharya et al., 2021), limonin (Vardhan and Sahoo, 2021), selenium, vitamins A, C and D, zinc, bioactive peptides (Galanakis et al., 2020), chitosan (Jaber et al., 2021), β-glucan, monolaurin (Günalan et al., 2021), arachidonic acid, docosahexaenoic acid and eicosapentaenoic acid (Das, 2020) was reported. Lycorine (IC50: 15.7 nM), an alkaloid from *Lycoris radiata* was efficient against SARS-CoV-1 (Li et al., 2005). Ilimaquinone from marine sponge demonstrated high affinity for various SARS-CoV-2 targets (3CLpro, PLpro, nsP10, nsP10, nsP10, nsP10, nsP10, 6MJ0J, and 7BTF) (Surti et al., 2020). Azadirachtin, a limonoid from the leaf extract of *Azadirachta indica Juss* stopped DV- 2 replication in suckling mice (Parida et al., 2002). Glycyrrhizin, a triterpene from liquorice roots, showed high antiviral activity in patients with SARS-CoV (Cinatl et al., 2003). Arachidonic acid and linoleic acid helped suppress MERS-CoV and HCoV-229E replication in infected cells (Yan et al., 2019). Regarding micronutrients, the supplementation of a bolus of vitamin D3 (before or during COVID-19 infection) to elderly people in a French nursing home increased the survival rate from 44.4% to 82.5% (Annweiler et al., 2020). Moderately and critically infected patients who received intravenous vitamin C doses remained hospitalized for 3–5 days, compared to 30 days of hospitalization for the other patients. In addition, none of the treated patients died (Cheng, 2020). Oral zinc supplementation was also reported to inhibit SARS-CoV-2 replication due to its interference and blockade with the S protein-ACE2 complex (McPherson et al., 2020). The use of fungal derivatives such as colosolactone VIII, E and G (Rangsinth et al., 2021) also showed anti-COVID-19 potential. Metabolites (mainly PCs) produced by *Aspergillus terreus* disrupted the 3CLpro activity of SARS-CoV-2 (El-Hawary et al., 2021). Other alternatives were also evaluated such as supplementation of zinc oxide nanoparticles (ZONs), which showed higher antiviral activity than hesperidin (Attia et al., 2021).

Synergistic use should also be evaluated. *In vitro*, Angelis et al. (2021) determined the activity of a mixture of ellagic acid, polydatin, honokiol, pterostilbene, selenium zinc and chromium against influenza A virus and SARS-CoV-2. For comparison purposes, the authors also evaluated the effect of polydatin alone. Polydatin (20 mg/L) inhibited the expression of hemagglutinin and nucleoprotein of influenza A virus by 28 and 35%, respectively, while the lower dose compound mixture (5 mg/L) had greater inhibition for both (≈45 and ≈40%, respectively). Regarding SARS-CoV-2, polydatin had no effect when used before or after infection; however, the mixture reduced the virus concentration by 1.8 and 2 logs at the different times, respectively. Biancatelli et al. (2020) determined that the combined use of quercetin and vitamin C, as opposed to their separate use, offers greater antiviral efficiency with a high potential for the current pandemic context.

Anti-COVID-19 therapy based on natural compounds should complement the drugs accepted and indicated by the health entities concerned. It is often a mistake to believe that natural compounds are sufficient, considering that this may be the case, but in, for example, for prevention or in cases of mild infection. Yildirim et al. (2016) analyzed the effect of propolis, acyclovir (drug), and their combined use against HSV-1 and HSV-2. HSV-1 replication was ≈4 × 10^7^, 2 × 10^7^ and 1.5 × 10^7^, when using propolis, acyclovir and propolis + acyclovir, respectively. Likewise, HSV-2 replication was 4.5 × 10^7^, 2 × 10^7^ and 1.5 × 10^7^, when using propolis, acyclovir and propolis + acyclovir, respectively, at 24 h. Further studies are needed to evaluate the synergy between various agents with confirmed properties of interest. To find out which compounds are under constant investigation, it is recommended to explore https://sbnb.irbbarcelona.org/covid19/. This platform is constantly updated and provides information on more than 1 million bioactive compounds with proven efficiency against COVID-19.

Another key point is that, it must be ensured that drugs are directed to different targets, which is often a limitation (Liu et al., 2022). PCs act against multiple SARS-CoV-2 targets in parallel and rapidly. Isoliquiritigenin and kaempferol from *Broussonetia papyrifera* showed a significant effect against 3CLpro and PLpro (Paraiso et al., 2020). PCs from propolis, green tea, garlic, turmeric, soybean and echinacea disrupted the function of S protein, ACE2, 3CLpro and RdRp (Keflie and Biesalski, 2021). Chrysin from honey has the potential to bind to ACE2 and to inhibit in parallel the activity of S protein (Abedi et al., 2021). Caffeic acid inhibited the activity of E and N protein of SARS-CoV-2 (Ali et al., 2022). Likewise, isoliquiritigenin and kaempferol from *Broussonetia papyrifera* inhibited 3CLpro and PLpro activity (Paraiso et al., 2020).

On the other hand, the use of plant matrices in particular (i.e., when isolated compounds are not used) is recommended because, in addition to polyphenols, they also contain other substances with biological activity. Therefore, synergistically, antiviral, antioxidant, anti-inflammatory and immunomodulatory effects would be enhanced (Chojnacka et al., 2020). However, Chapman and Andurkar (2022) mention that this is a double-edged sword. The argument is that the mixture of compounds can also have an antagonistic effect on each other. Another option is the use of isolated compounds, but the process of identification, separation and purification is complex. Moreover, at this last point, the compounds may partially or even totally lose their biological activity. Studies are suggested to help explore this field in depth in order to solve the aforementioned problem.

There is still much to be explored with respect to the evaluation of efficient compounds against COVID-19. More clinical trials are needed to provide information on other PCs and plant matrices rich in PCs that have not yet been explored. Although the current outlook is discouraging, the challenges create an opportunity to improve the natural medicine system (Wakeman et al., 2020). When this crisis is overcome, there will be a wide range of options to counteract not only viral diseases, but also other diseases of global concern.

## 3 Conclusion

The use of bioactive compounds is being extensively investigated, highlighting PCs to deal with COVID-19 due to its known high antiviral activity. According to the studies evaluated, polyphenols have shown an efficient activity against SARS-CoV-2. This is because PCs act on proteins of the virus, interfering in its different mechanisms of infection. In addition, PCs consumption helps modulate the immune system through several mechanisms of action, which significantly influences the prevention against COVID-19, mainly avoiding the appearance of severe symptoms. *In silico, in vitro* and *in vivo* studies have allowed us to determine the anti-COVID-19 potential and mechanism of action of PCs. Clinical trials have demonstrated the effectiveness of PCs in the prevention and as a possible therapeutic management against COVID-19, ruling out adverse effects. It is also recommended to explore new compounds and drugs with proven antiviral activity to test their individual and synergistic efficacy with PCs against SARS-CoV-2.

## Declarations

### Author contribution statement

All authors listed have significantly contributed to the development and the writing of this article.

### Funding statement

This research did not receive any specific grant from funding agencies in the public, commercial, or not-for-profit sectors.

### Data availability statement

Data included in article/supp. material/referenced in article.

### Declaration of interest’s statement

The authors declare no conflict of interest.

### Additional information

No additional information is available for this paper.
